# Safe practices for prevention and management of antineoplastic agent extravasation: development of an educational video

**DOI:** 10.1590/0034-7167-2024-0172

**Published:** 2024-11-22

**Authors:** Rafael Fernando Mendes Barbosa, Anne Kettley Lacera de Lima Gonzaga, Fabrine Aguilar Jardim, Karina Dal Sasso Mendes, Namie Okino Sawada

**Affiliations:** I Universidade do Estado de Minas Gerais. Passos, Minas Gerais, Brazil; II Universidade de São Paulo. Ribeirão Preto, São Paulo, Brazil; III Universidade Federal de Alfenas. Alfenas, Minas Gerais, Brazil

**Keywords:** Nursing, Antineoplastic Agents, Extravasation of Diagnostic and Therapeutic Materials, Instructional Film and Video, Educational Technology, Enfermería, Antineoplásicos, Extravasación de Materiales Terapéuticos y Diagnósticos, Película y Video Educativos, Tecnología Educacional

## Abstract

**Objectives:**

to develop, validate, and evaluate an educational video on the prevention and management of antineoplastic agent extravasation, aimed at nursing professionals.

**Methods:**

this methodological study was developed according to Falkembach’s theoretical framework, which outlines five phases in the production of educational video materials: analysis and planning, modeling, implementation, evaluation, and distribution.

**Results:**

content validation demonstrated agreement above the minimum threshold stipulated. The overall Content Validity Index was 90.8%, and it was 94.2% among the content and technical evaluation judges, respectively. The target audience evaluated the video positively, highlighting the importance of the content, the clarity of the language used, and the understanding of the information pertinent to the topic.

**Conclusions:**

the video proved to be an appropriate strategy for instructing interventions on the prevention and management of extravasation, with the potential to improve educational practices among nursing professionals.

## RESUMEN

**Objetivos:**

construir, validar y evaluar un video educativo sobre la prevención y el manejo de la extravasación de agentes antineoplásicos, dirigido a profesionales de enfermería.

**Métodos:**

estudio de naturaleza metodológica, desarrollado según el referencial teórico de Falkembach, que preconiza cinco fases en la producción de materiales educativos en video: análisis y planificación, modelado, implementación, evaluación y distribución.

**Resultados:**

la validación de contenido demostró una concordancia por encima del umbral mínimo estipulado. El Índice de Validez de Contenido general fue del 90,8% y del 94,2% entre los jueces de evaluación de contenido y de técnica, respectivamente. El público objetivo evaluó el video de manera positiva, destacando la importancia del contenido, la claridad del lenguaje utilizado y la comprensión de la información pertinente a la temática.

**Conclusiones:**

el video demostró ser una estrategia adecuada para instruir intervenciones sobre la prevención y el manejo de la extravasación, con el potencial de mejorar las prácticas educativas entre profesionales de enfermería.

## INTRODUCTION

The extravasation of antineoplastic agents is described as the inadvertent leakage of vesicant substances from within the blood vessel into surrounding tissues. This occurrence can result in painful sensations, edema, hyperemia, tissue damage, vesicle formation, skin peeling, tissue necrosis, and considerable morbidity. In many cases, it may even require surgical intervention and frequently results in restrictive sequelae^(1,2)^. The spread of these chemical substances constitutes a genuine oncological emergency. Due to the unique mechanism of action of antineoplastic agents, the implications of their occurrence, as well as their consequences and appropriate management, are widely discussed topics in the scientific literature^(3-5)^.

Studies on the knowledge of nursing professionals regarding the extravasation of antineoplastic agents have revealed a considerable number of professionals working in chemotherapy who are unable to accurately describe, or are even unaware of, the recommended sequence for managing an extravasation episode, evidencing a lack of knowledge about the care required in such adverse events^(6-8)^.

Thus, it is essential that nursing professionals involved in cancer care, particularly in the administration of antineoplastic agents, possess specific knowledge about the adverse events associated with the management of the extravasation of these chemical substances^(6,7,9)^. Their professional performance demands mastery of theoretical knowledge, competencies, technical skills, and updated practices, as this ensures the provision of safe and effective nursing care^(7,8)^.

Considering the incorporation of technological resources in the training process of nursing professionals focused on managing the care of people with cancer undergoing chemotherapy, to ensure the provision of systematic, effective, and high-quality care, the development of educational material in video format emerges as a viable teaching-learning strategy^(10,11)^.

The development of an educational video not only equips, strengthens, and stimulates the understanding of the target audience but also provides support for oncology nursing practice. This type of resource plays a significant role as a teaching tool in the professional training of nurses and additionally enhances the quality of nursing care for cancer patients^(12)^. Furthermore, it is noteworthy that this approach constitutes a relevant tool for the dissemination and promotion of Evidence-Based Practice.

Although studies that evaluate the knowledge and performance of nurses regarding the prevention and management of antineoplastic agent extravasation, considering the complexity of chemotherapy treatment, are available in Brazil and worldwide, there remains a knowledge gap and a deficiency in the proper execution of management strategies^(6-8)^.

Notably, to date, the literature does not present any research dedicated to the development of an educational video on the extravasation of antineoplastic agents, only manuals^(2)^ and other educational materials^(13)^. In view of this, the need for a continuous, attractive educational approach that is easily accessible and has a broad geographic reach, such as an educational video directed at these professionals, becomes undeniable. This would fill the identified gap and ensure enduring educational practice.

## OBJECTIVES

To develop, validate, and evaluate an educational video on the prevention and management of antineoplastic agent extravasation, aimed at nursing professionals.

## METHODS

### Ethical aspects

Ethical principles were rigorously followed according to Resolution 466/12. The research was approved by the Ethics Committee with the Certificate of Presentation for Ethical Consideration (CAAE). The professionals involved in the video signed the Image and Voice Use Authorization Terms, while the content and technical evaluation judges and the nurses provided their consent through the Free and Informed Consent Term. It is worth noting that the contributions of the actors and the narrator were voluntary.

### Study design, period, and location

This is a methodological study^(14)^ encompassing the pre-production (elaboration and validation of the script and storyboard), production (video recording based on the validated script and storyboard), and post-production (video evaluation by the target audience) stages. It was developed according to Falkembach’s theoretical framework^(15)^, which prescribes five phases in the production of educational video materials: analysis and planning, modeling, implementation, evaluation, and distribution.

The educational video was created at a public university located in the city of Ribeirão Preto (SP, Brazil). The complete process of creating the educational video encompassed all the previously outlined stages and spanned a total period of 28 months, from February 2021 to June 2023.

The content of the script and storyboard was prepared in accordance with international guidelines^(16)^ and available evidence in the literature regarding the prevention and management practices for antineoplastic agent extravasation^(17)^, with the purpose of incorporating the best evidence on the topic and supporting the construction of the script and storyboard, following the five phases of Falkembach’s theoretical framework^(15)^.

Thus, in the analysis and planning phase, a thorough analysis of the bibliographic research on the topic was conducted. During this phase, the topic of interest, along with similar applications and available resources, was evaluated and defined.

The modeling phase allowed for the construction of the script and storyboard through the creation of models aimed at facilitating understanding, promoting discussion, and obtaining approval of a system before its effective implementation. In the application stage, three types of models were conceived: conceptual, navigation, and interface. In the first, aspects for the development of the content to be made available were observed. The navigation model considered the organization of the material according to indices, menus, and user guides. The interface created the visual identity of the product, defining a set of elements that presented the organization of information and user actions.

The implementation stage included the production, reuse, and digitization of the media through the creation of the project’s media, including sounds, images, and animations, using specific software, based on the validated script and storyboard.

The evaluation and maintenance phase allowed the target audience to view the educational video, and finally, the distribution phase involved defining the method to execute and disseminate the educational video. In this study, after the recordings and respective edits were completed, the educational video was made available through a globally recognized international web page.

### Sample, inclusion, and exclusion criteria

For content validation, an instrument with questions related to objectives, content, relevance, and environment was used, and for technical validation, another instrument composed of questions related to functionality, usability, and efficiency was utilized. These evaluation instruments were based on an adaptation of a previously employed method^(18)^. For the target audience evaluation, an instrument was developed to assess the understanding of the educational video information on the topic in question.

Thus, for content validation, the instrument was presented to nine judges recruited through their profiles on the Lattes Platform, considering their doctoral degrees and specialized knowledge on the subject under study, demonstrated by their specialist titles, professional experience, and published research in the relevant area.

Technical validation was carried out by five judges with a minimum of two years of experience in Digital Information and Communication Technologies, with an emphasis on video production. Additionally, these professionals specialized in digital technologies in health and nursing and worked at the university where the study was conducted.

After the suggested adjustments and to assess operational adequacy, the educational video was reviewed by the target audience, which consisted of nurses working in the care of people with cancer undergoing chemotherapy. Their profile closely matched the target audience for which the educational video was developed. These nurses were randomly selected and recommended by the researcher based on their competencies and relevant professional experience.

The initial approach to the judges was made through an electronic invitation letter explaining the study. Those who agreed to participate as judges received the Free and Informed Consent Term. Subsequently, the script and storyboard of the educational video were made available to these participants, along with a link to access the evaluation instrument through an online platform.

### Data analysis and statistics

For reliability analysis, the Content Validity Index (CVI) was calculated for each item of the instruments. An agreement of 78% was considered validated for each domain of the instruments used by the content and technical evaluation judges, as for six or more judges, a minimum CVI of 78% is recommended^(19)^. All items on the instrument were organized in a checklist format, with options for each item marked as: “disagree,” “partially disagree,” “neutral (neither agree nor disagree),” “partially agree,” and “agree,” on a Likert scale of one to five, with space for justification/comment in all variables. Thus, the instrument could generate an adherence score obtained from the following formula:


$$\mathrm{CVI}=\left(\frac{n_{\text{agree }}+n_{\text{partially agree }}}{n_{\text{total }}}\right)\times 100$$

Where

- nagree is the number of "agree" responses.
- npartially agree is the number of "partially agree" responses.
- ntotal is the total number of responses.

This formula calculates the CVI as a percentage, reflecting the proportion of judges who agreed or partially agreed with the item being validated. Items that did not meet this threshold were reviewed and adjusted based on the feedback provided by the judges. This rigorous validation process ensures that the instrument is reliable and accurately measures the intended content.

In this study, the CVI was calculated for each domain and overall, allowing for a comprehensive assessment of the instrument’s validity. The overall CVI provides a holistic measure of the instrument’s reliability, while the domain-specific CVIs highlight areas of strength and potential improvements.

## RESULTS

The final production of the educational video has a duration of 18 minutes and 18 seconds, comprising narrative and simulated scenes to provide a better understanding of the logical sequence of events. The video includes a conceptual approach and risk factors regarding extravasation for nurses; reception, setup of the chemotherapy regimen, and prevention actions; guidance on and clinical manifestations of extravasation; and appropriate management in the event of extravasation. Additionally, the video provides subtitles as an additional technological tool to enhance the target audience’s understanding, thereby contributing to the optimization of high-quality nursing care.

The educational video, titled “Prevention and Management of Antineoplastic Agent Extravasation,” is available online at: https://www.youtube.com/watch?v=02pGCHrndns. The subtitles aim to improve the comprehension of the target audience, thus enhancing the delivery of high-quality nursing care.

The judges involved in the validation of the content of the script and storyboard of the educational video were all nursing professionals with doctoral degrees and proven prior experience in the administration of antineoplastic drugs. They evaluated the script and storyboard, making suggestions regarding relevance and environment. They highlighted that some terminologies and images should be replaced or supplemented, as they were considered inadequate.

The average and overall CVI resulting from the evaluation exceeded the minimum agreement threshold stipulated for this study. Specifically, the overall CVI reached 90.8%, reaffirming the significant contribution of the educational video to the enrichment of the knowledge area (Table 1).

**Table 1:** Distribution of absolute frequency of the agreement level of content evaluation judges (n = 9) and **Content Validity Index** of each domain of the validation instrument for the script and storyboard of the educational video, Ribeirão Preto, São Paulo, Brazil, 2024

Domain	D^†^	PD^‡^	N^§^	PA^ǁ^	A^¶^	CVI^*^(%)
Objectives						
The objectives are consistent with nursing practice.	-	-	-	1	8	100
The objectives are appropriate to be achieved.	-	-	-	-	9	100
Mean CVI	-	-	-	-	-	100
Content						
The content presented in the script and storyboard corresponds to the proposed objectives.	-	-	-	3	6	100
The content facilitates the teaching-learning process regarding the prevention and management of antineoplastic agent extravasation.	-	-	-	2	7	100
The content allows the understanding of prevention and management of antineoplastic agent extravasation.	-	-	-	3	6	100
The content follows a logical sequence.	-	-	-	2	7	100
The content incorporates the necessary steps for the prevention and management of antineoplastic agent extravasation.	-	-	-	4	5	100
The content includes the necessary materials for demonstrating the prevention and management of antineoplastic agent extravasation.	-	-	-	1	8	100
The information in the script and storyboard is correct.	1	-	-	3	5	89
Mean CVI	-	-	-	-	-	98.4
Relevance						
The images and scenes illustrate important aspects for the practice of prevention and management of antineoplastic agent extravasation.	-	-	2	2	5	78
The images and scenes are relevant for nurses to understand the prevention and management of antineoplastic agent extravasation.	-	-	1	2	6	89
The images and scenes allow the transfer and use of theoretical/practical knowledge in different contexts by nurses.	-	-	2	1	6	78
Mean CVI	-	-	-	-	-	81.6
Environment						
The setting is adequate for video production.	-	-	2	2	5	78
The setting is adequate for teaching and learning about the prevention and management of antineoplastic agent extravasation.	-	-	1	2	6	89
Mean CVI	-	-	-	-	-	83.5
Overall CVI	-	-	-	-	-	90.8

* *- Content Validity Index; † - Disagree; ‡ - Partially Disagree; § - Neutral;* ǁ *- Partially Agree; ¶ - Agree.*

Among the technical evaluation judges, all worked in the field of communication and audiovisual technologies with an emphasis on health and nursing, with one holding a master’s degree, three with specializations, and one with a bachelor’s degree. In the validation of the script and storyboard technique, there was 100% agreement in the domain related to functionality. The usability and efficiency domains obtained a minimum agreement of 88%, justified by the judges who noted that the visual language could help clarify some points and make the content more dynamic. Regarding efficiency, one judge highlighted the need to include more chemotherapy regimens in the video, a suggestion that was not adopted due to the recommended duration limitation for educational videos.

The average and overall CVI reached the minimum required agreement level, presenting an overall CVI of 94.2%, as observed in Table 2.

**Table 2:** Distribution of absolute frequency of the agreement level of technical evaluation judges in video (n = 5) and Content Validity Index of each domain of the validation instrument for the script and storyboard of the educational video, Ribeirão Preto, São Paulo, Brazil, 2024

Domain	D^†^	PD^‡^	N^§^	PA^ǁ^	A^¶^	CVI^*^(%)
Functionality						
The script and storyboard of the video propose comprehensible nursing interventions for the prevention and management of antineoplastic agent extravasation.	-	-	-	3	2	100
The script and storyboard of the video have the potential to generate positive results.	-	-	-	-	5	100
Mean CVI	-	-	-	-	-	100
Usability						
It is easy to learn the concepts that will be used in the video and their applications.	-	1	-	2	2	80
The video will enable nurses to learn the interventions for the prevention and management of antineoplastic agent extravasation.	-	-	-	2	3	100
The video will assist nurses clearly and efficiently without being tiresome.	-	1	-	1	3	80
Mean CVI	-	-	-	-	-	86.7
Efficiency						
The proposed time is adequate for the user to learn the content.	-	-	-	2	3	100
The number of scenes is consistent with the proposed time for the video.	-	-	-	2	3	100
The number and characterization of the characters meet the proposed objective.	-	-	-	1	4	100
Communication between the characters occurs efficiently and comprehensibly.	-	-	-	3	2	100
The description of the materials to be used is clear.	1	-	-	1	3	80
Mean CVI	-	-	-	-	-	96
Overall CVI	-	-	-	-	-	94.2

* *- Content Validity Index; † - Disagree; ‡ - Partially Disagree; § - Neutral;* ǁ *- Partially Agree; ¶ - Agree.*

Regarding the evaluation of the educational video by the target audience, a total of 20 nurses were invited to participate, of whom eleven accepted the invitation. Among them, seven professionals worked in a chemotherapy outpatient clinic, three in an oncology inpatient unit, and one in a Bone Marrow Transplant (BMT) Unit, with professional experience ranging from one to 20 years, with an average of nine years. As for academic qualifications, nine had specializations, one had a doctorate, and one had only a bachelor’s degree.

Table 3 demonstrates the aspects of the evaluation of the educational video by the target audience, indicating that most evaluated the video positively. Only one professional selected the “disagree” option.

**Table 3:** Distribution of the target audience’s responses regarding the evaluation of the educational video, Ribeirão Preto, São Paulo, Brazil, 2023

Variable	Yes		No	
	f	%	f	%
Does the video content present important information for the practice of prevention and management of antineoplastic agent extravasation?	11	100	-	-
Is the video content clear and understandable?	11	100	-	-
Do the images and scenes help in understanding the extravasation of antineoplastic agents?	11	100	-	-
Does watching the video help in understanding the extravasation of antineoplastic agents?	11	100	-	-
Is the video duration adequate?	10	90.9	1	9.1

The participants evaluated the video positively, noting that the content presents significant information with clarity and language that enhances the understanding of the topic. The suggestions provided by the participants were reviewed and incorporated as appropriate. Consequently, the video was validated with the potential to contribute significantly to the teaching of the proposed topic, as also indicated by the opinions of the content and technical evaluation judges.

## DISCUSSION

All oncology team professionals share the responsibility of ensuring the safe administration of chemotherapy^(9)^. However, the nursing team is subject to more intense demands due to the potential injuries resulting from the extravasation of these agents. Consequently, it is imperative that nursing professionals are not only aware of the inherent risks but also possess technical skills, understand the importance of these procedures, and avoid distractions and interruptions during the administration of chemotherapy^(6-8)^.

The development of additional educational resources, such as the video, emerges as an approach that strengthens the integration of evidence-based interventions while offering a technological solution that enhances health education. The video, with its attractive features and broad accessibility due to easy availability, can reach a wide geographic audience, reinforcing its utility as an effective means of disseminating knowledge^(20)^.

Therefore, the intrinsic relevance of this study is evident with the creation of an educational video aimed at providing a fundamental resource to enhance the training of nurses in the care of cancer patients, focusing on the prevention and management of antineoplastic agent extravasation. In developing the educational video, significant attention was given to creating a simulated environment resembling the scenarios and material resources found in outpatient chemotherapy services, including a private space for the care of cancer patients. Studies in nursing have revealed the growing use of clinical simulation as an active teaching methodology in health^(21-23)^.

The production of an educational video is a rigorous, long-term, and intense process, demanding considerable effort from the researchers. Its objective is to create a tool enriched with scientifically based interventions that promote the construction of knowledge according to the needs of nursing professionals. This endeavor follows a rigorous approach, ensuring the creation of solid and evidence-based digital educational material^(24)^.

In the present study, the educational video was developed based on the preparation of a script and storyboard, using a fictitious clinical case that integrates the primary researcher’s professional experience. Additionally, knowledge gaps^(6-8)^ in nursing care related to the prevention and management of antineoplastic agent extravasation were investigated.

The use of a script and storyboard in the development of an educational video is an approach that encompasses the sequential organization of the content to be produced, including the analysis of the scenario, available resources, and materials. This strategy aims to specify details such as narration, images, and scenes involved in the production of educational videos^(25)^.

Studies have revealed the use of digital educational materials, such as videos in the context of nursing, with the advanced construction of the script and storyboard, similar to the production carried out in this study. These studies demonstrate that the use of a script and storyboard makes the creation process more structured, facilitating the target audience’s understanding of the topic. Such research has relied on clinical cases experienced in various health settings, grounded in knowledge for the creation of educational materials^(20,26-28)^.

The validation of a script and storyboard requires the careful selection of judges in the subject matter under study. In this study, the criteria established by Fehring^(29)^, a widely recognized approach that aims at the utility of the instrument for application in health practice, were adopted.

The selection of content judges through the CNPq Lattes Platform is a strategy frequently used by researchers, allowing the identification of professionals working in different health realities in the country. This approach helps to gather valuable perspectives, especially concerning the nuances of care in different local contexts of interest^(26,30,31)^.

The validation of the script and storyboard reached the minimum agreement level required for this study, revealing a result similar to other educational video development studies^(20,26-28)^. The evaluation of the video by the target audience was crucial to confirm the validation made by the expert judges.

Although suggestions for changes in the script, storyboard, and the video itself were presented by the content and technical evaluation judges and the target audience, these considerations resulted in improvements in the coherence of the video’s elements. As a result, the educational video was validated as a valuable tool to enhance the teaching of the proposed topic.

However, even after the entire process of validation, recording, editing, and finalizing the video, some weaknesses were identified, such as the use of a common gown (contact isolation type) when the recommended gown is impermeable with tight cuffs to prevent skin exposure^(16)^; the professional’s failure to maintain a postural position during the infusion of vesicant chemotherapeutic agents, making it impossible to evaluate the puncture site and the clinical and emotional manifestations of extravasation in the patient^(16)^; and finally, the use of a single pair of gloves, while evidence advises using two pairs of procedure gloves since there are micro holes in this type of glove, and one over the other would avoid the primary route of contamination for the professional, which is dermatological^(32)^.

The final version of the educational video exceeded the recommended duration of 15 minutes, which may cause viewer distraction, according to previous studies^(25,33)^. However, there are divergences regarding the ideal duration limit for educational videos aimed at health, varying between 15 to 20 minutes^(34)^, which raises questions about the recommendation to follow.

In summary, the educational video was made available online through a digital platform, enabling audiovisual communication on the Internet, as proposed by Falkembach^(15)^, through the distribution phase. The choice of this environment was justified by ease of access and the interest in reinforcing, in a didactic way, the guidance provided to nursing professionals on the topic.

### Study limitations

Despite the positive results and the evaluation of the educational video, it is important to mention some limitations of this study, such as the size of the script and storyboard, which may have impacted the specialists’ evaluation, the final duration of the video, the lack of subtitles in other languages, and resources to teach Libras to people with hearing impairments. However, the positive evaluation of the video is encouraging. For future studies, it is recommended to apply experimental research to assess its effectiveness and use in clinical nursing practice as a health education tool. This is a new objective for subsequent investigations.

### Contributions to the Field of Nursing

This study offers a valuable contribution by providing an innovative educational tool for nursing professionals, addressing the topic in an accessible manner on freely accessible digital platforms. This has the potential to benefit the care of cancer patients by overcoming geographical barriers. The research highlights how technologies can drive progress in nursing knowledge, playing crucial roles in both education and care.

## CONCLUSIONS

The construction, validation, and evaluation of the educational video represented an effective and well-received approach by both content and technical judges as well as the target audience. The video proved to be an appropriate strategy for instructing interventions on the prevention and management of antineoplastic agent extravasation, with the potential to improve educational practices among nursing professionals. The results achieved the established objectives and presented adequate validation indices. Furthermore, this process highlighted the empowerment of nursing praxis in creating effective digital educational materials, revealing new perspectives in the organization and management of care.
