# Supplementary material for: Safe practices for prevention and management of antineoplastic agent extravasation: development of an educational video
Source: Rev Bras Enferm. 2024 Nov 22;77(5):e20240172. doi: 10.1590/0034-7167-2024-0172 (PMC11653873; doi:10.1590/0034-7167-2024-0172)
Supplement: Translation

# Translation: Práticas seguras para prevenção e manejo do extravasamento de agentes antineoplásicos: desenvolvimento de vídeo educativo

**DOI:** 10.1590/0034-7167-2024-0172pt

**Authors:** Rafael Fernando Mendes Barbosa, Anne Kettley Lacera de Lima Gonzaga, Fabrine Aguilar Jardim, Karina Dal Sasso Mendes, Namie Okino Sawada

**Affiliations:** I Universidade do Estado de Minas Gerais. Passos, Minas Gerais, Brasil; II Universidade de São Paulo. Ribeirão Preto, São Paulo, Brasil; III Universidade Federal de Alfenas. Alfenas, Minas Gerais, Brasil

**Keywords:** Enfermagem, Antineoplásicos, Extravasamento de Materiais Terapêuticos e Diagnósticos, Filmes e Vídeos Educativos, Tecnologia Educacional

## Abstract

**Objetivos:**

construir, validar e avaliar um vídeo educativo sobre a prevenção e o manejo do extravasamento de agentes antineoplásicos, direcionado a profissionais de enfermagem.

**Métodos:**

estudo de natureza metodológica, desenvolvido segundo o referencial teórico de Falkembach, que preconiza cinco fases na produção de materiais educativos em vídeo: análise e planejamento, modelagem, implementação, avaliação e distribuição.

**Resultados:**

a validação de conteúdo demonstrou concordância acima do limiar mínimo estipulado. O Índice de Validade de Conteúdo geral foi de 90,8% e 94,2% entre os juízes de avaliação de conteúdo e de técnica, respectivamente. O público-alvo avaliou o vídeo de maneira positiva, destacando a importância do conteúdo, a clareza da linguagem utilizada e a compreensão das informações pertinentes à temática.

**Conclusões:**

o vídeo demonstrou ser uma estratégia adequada para instruir intervenções sobre a prevenção e o manejo do extravasamento, com potencial para melhorar as práticas educativas entre profissionais de enfermagem.

## INTRODUÇÃO

O extravasamento de agentes antineoplásicos é descrito como a fuga inadvertida de substâncias vesicantes do interior do vaso sanguíneo para tecidos circundantes. Essa ocorrência pode resultar em sensações dolorosas, edema, hiperemia, comprometimento tissular, formação de vesículas, descamação cutânea, necrose tecidual e considerável morbidade. Em muitos casos, pode inclusive requerer intervenção cirúrgica e frequentemente resulta em sequelas de natureza restritiva^(1,2)^. A disseminação dessas substâncias químicas assume a forma de uma autêntica emergência oncológica. Devido à singularidade do mecanismo de ação dos agentes antineoplásicos, as implicações de sua ocorrência, bem como suas consequências e a gestão apropriada, são temas amplamente discutidos na literatura científica^(3-5)^.

Estudos sobre o conhecimento de profissionais de enfermagem, no que se refere ao extravasamento de agentes antineoplásicos, têm revelado um índice considerável de profissionais atuantes na área da quimioterapia que não demonstram a capacidade de descrever de forma precisa, ou até mesmo desconhecem a sequência recomendada para o manejo a ser adotado em face de um episódio de extravasamento, evidenciando desconhecimento dos cuidados dispensados na ocorrência deste evento adverso^(6-8)^.

Assim, é imprescindível que os profissionais de enfermagem engajados na assistência à pessoa com câncer, com foco particular na administração de agentes antineoplásicos, detenham conhecimentos específicos acerca dos eventos adversos vinculados à gestão do extravasamento dessas substâncias químicas^(6,7,9)^. Sua atuação profissional demanda um domínio de conhecimentos teóricos, competências, habilidades técnicas e prática atualizada, uma vez que isso assegura a oferta de uma assistência de enfermagem segura e efetiva^(7,8)^.

Ao considerar a incorporação de recursos tecnológicos no processo de capacitação dos profissionais de enfermagem voltados para o gerenciamento da assistência à pessoa com câncer em tratamento quimioterápico, a fim de garantir a provisão de uma assistência sistematizada, efetiva e de alta qualidade, a construção de um material educativo em formato de vídeo emerge como uma estratégia viável de ensino-aprendizagem possível de ser utilizada^(10,11)^.

O desenvolvimento de vídeo educativo não apenas instrumentaliza, fortalece e estimula a compreensão do público-alvo a que se destina, mas também fomenta subsídios para a prática de enfermagem oncológica. Esse tipo de recurso desempenha um papel significativo como ferramenta de ensino na formação profissional do enfermeiro e, adicionalmente, incrementa a qualidade da assistência de enfermagem aos pacientes com câncer^(12)^. Além disso, merece destaque o fato de que essa abordagem constitui uma ferramenta relevante para a disseminação e promoção da Prática Baseada em Evidências.

Embora estudos que avaliem o conhecimento e a atuação de enfermeiros sobre a prevenção e o manejo do extravasamento de agentes antineoplásicos, diante da complexidade do tratamento quimioterápico, estejam disponíveis no Brasil e no mundo, persiste uma lacuna de conhecimento e uma deficiência na execução adequada das estratégias de manejo^(6-8)^.

Notavelmente, até o presente momento, a literatura não apresenta nenhuma pesquisa que tenha se dedicado à elaboração de um vídeo educativo sobre o extravasamento de agentes antineoplásicos, mas somente de manuais^(2)^ e outros materiais educativos^(13)^. Em vista disso, torna-se inegável a necessidade de uma abordagem educativa contínua, atrativa, de fácil acesso e de grande alcance geográfico como o vídeo educativo, direcionada a esses profissionais, a fim de preencher essa lacuna identificada e garantir uma prática educacional duradoura.

## OBJETIVOS

Construir, validar e avaliar um vídeo educativo sobre a prevenção e manejo do extravasamento de agentes antineoplásicos, direcionado a profissionais de enfermagem.

## MÉTODOS

### Aspectos éticos

Os princípios éticos foram rigorosamente seguidos, de acordo com a Resolução 466/12. A pesquisa foi aprovada pelo Comitê de Ética com o Certificado de Apresentação de Apreciação Ética (CAAE). Os profissionais envolvidos no vídeo assinaram os Termos de Autorização de Uso da Imagem e Voz, enquanto os juízes de avaliação de conteúdo e de técnica e os enfermeiros deram seu consentimento através do Termo de Consentimento Livre e Esclarecido. Vale ressaltar que as contribuições dos atores e da locutora foram voluntárias.

### Desenho, período e local e do estudo

Estudo de natureza metodológica^(14)^, abrangendo as etapas de pré-produção (elaboração e validação do roteiro e *storyboard)*, produção (gravação do vídeo a partir do roteiro e *storyboard* validados) e pós-produção (avaliação do vídeo pelo público-alvo), desenvolvido segundo referencial teórico de Falkembach^(15)^, que preconiza cinco fases na produção de materiais educativos em vídeo: análise e planejamento, modelagem, implementação, avaliação e distribuição.

O vídeo educativo foi elaborado nas dependências de uma universidade pública localizada na cidade de Ribeirão Preto (SP, Brasil). O processo completo de criação do vídeo educativo englobou todas as etapas anteriormente delineadas e estendeu-se por um período total de 28 meses. Esse intervalo temporal compreendeu o período de fevereiro de 2021 a junho de 2023.

O conteúdo do roteiro e do *storyboard* foi elaborado em consonância com as recomendações de diretrizes internacionais^(16)^ e de evidências disponíveis na literatura acerca das práticas de prevenção e manejo no extravasamento de agentes antineoplásicos^(17)^, com o propósito de incorporar as melhores evidências sobre a temática e embasar a construção do roteiro e do *storyboard*, obedecendo as cinco fases do referencial teórico de Falkembach^(15)^.

Assim, na fase de análise e planejamento, procedeu-se à análise minuciosa da pesquisa bibliográfica encontrada sobre a temática. Nesta fase, o tema de interesse, juntamente com as aplicações similares e os recursos disponíveis, foram avaliados e definidos.

A fase de modelagem permitiu a construção do roteiro e do *storyboard*, através da criação de modelos, com o objetivo de facilitar a compreensão, promover a discussão e obter a aprovação de um sistema antes da sua efetiva implementação. Na etapa de aplicação, foram concebidos três tipos de modelos: conceitual, de navegação e de interface. No primeiro, foi observado os aspectos para a elaboração do conteúdo que seria disponibilizado. O modelo de navegação observou a organização do material, de acordo com índices, menus e guias de orientação para o usuário. A interface criou a identidade visual do produto, definindo um conjunto de elementos que apresentasse a organização das informações e as ações do usuário.

A etapa de implementação abrangeu a produção, reutilização e digitalização das mídias, através da criação das mídias do projeto, incluindo os sons, as imagens e animações, utilizando softwares específicos, a partir do roteiro e *storyboard* validados.

A fase de avaliação e manutenção, permitiu a visualização do vídeo educativo, pelo público-alvo, e por fim, a fase de distribuição envolveu a definição do método para executar e disseminar o vídeo educativo. Neste estudo, após a finalização das gravações e das respectivas edições, o vídeo educativo foi disponibilizado por meio de uma página *web* de alcance internacional, mundialmente reconhecida.

### Amostra, critérios de inclusão e exclusão

Para a validação do conteúdo, foi utilizado um instrumento com questões referentes aos itens objetivo, conteúdo, relevância e ambiente e, para a validação técnica, utilizou-se outro instrumento composto por questões referentes aos itens funcionalidade, usabilidade e eficiência. Esses instrumentos de avaliação foram baseados em uma adaptação de um método empregado previamente^(18)^. Já para a avaliação do público-alvo, foi elaborado um instrumento referente à compreensão das informações do vídeo educativo sobre a temática em questão.

Assim, para a validação de conteúdo, o instrumento foi apresentado à apreciação de nove juízes, recrutados por meio do currículo na Plataforma Lattes, considerando a titulação (doutor), além do conhecimento especializado sobre o assunto em estudo, demonstrado por meio de título de especialista, experiência profissional e pesquisas publicadas na área em análise.

A validação técnica foi realizada por cinco juízes convidados com experiência igual ou superior a dois anos na área de Tecnologias Digitais de Informação e Comunicação, com ênfase em produção de vídeo. Além disso, esses profissionais eram especializados em tecnologias digitais na área da saúde e enfermagem, e exerciam suas atividades nas dependências da universidade onde o estudo foi desenvolvido.

Após os ajustes sugeridos e para avaliar a adequabilidade operacional, o vídeo educativo passou pela apreciação do público-alvo, que consistiu em enfermeiros atuantes na assistência à pessoa com câncer em tratamento quimioterápico, cujo perfil refletia semelhança com a audiência-alvo para a qual o vídeo educativo estava sendo desenvolvido. Esses enfermeiros foram escolhidos de maneira aleatória e indicados pelo pesquisador, com base em suas competências e experiências no campo profissional relevante.

A abordagem inicial dos juízes ocorreu por meio de carta-convite encaminhada eletronicamente, com explicações sobre o estudo. Aqueles que concordaram em colaborar na qualidade de juízes receberam o Termo de Consentimento Livre e Esclarecido. Em seguida, foi disponibilizado para esses participantes o roteiro e o *storyboard* do vídeo educativo, juntamente com um *link* para acessar o instrumento de avaliação através de uma plataforma *online*.

### Análise dos dados e estatística

Para a análise de confiabilidade, foi calculado o Índice de Validade de Conteúdo (IVC) para cada item dos instrumentos. Considerou-se validada a concordância de 78% para cada domínio dos instrumentos utilizados pelos juízes de avaliação de conteúdo e de técnica, pois para seis ou mais juízes, é recomendado um IVC mínimo de 78%^(19)^. Todos os itens do instrumento foram organizados no modelo checklist, com as alternativas para marcação de cada item como: “discordo”, “discordo parcialmente”, “indiferente (nem concordo, nem discordo)”, “concordo parcialmente” e “concordo”, em uma escala Likert de um a cinco, com espaço para justificativa/comentário em todas as variáveis. Assim, o instrumento pode gerar um escore de adesão obtido da seguinte fórmula:


$$\mathrm{IVC}=\left(\frac{n_{\text{concordo }}+n_{\text{concordo parcialmente }}}{n_{\text{total }}}\right)\times 100$$

Onde:

- *nconcordo* é o número de respostas “concordo”.
- *nconcordo parcialmente* parcialmente é o número de respostas “concordo parcialmente”.
- *ntotal* é o número total de respostas.

Esta fórmula calcula o IVC como uma porcentagem, refletindo a proporção de juízes que concordaram ou concordaram parcialmente com o item sendo validado. Itens que não atingiram esse limite foram revisados e ajustados com base no feedback fornecido pelos juízes. Este rigoroso processo de validação garante que o instrumento seja confiável e meça com precisão o conteúdo pretendido.

Neste estudo, o IVC foi calculado para cada domínio e de forma geral, permitindo uma avaliação abrangente da validade do instrumento. O IVC geral fornece uma medida holística da confiabilidade do instrumento, enquanto os IVCs específicos de domínio destacam áreas de força e possíveis melhorias.

## RESULTADOS

A produção final do vídeo educativo possui duração de 18 minutos e 18 segundos, constituído por meio de narrativa e cenas simuladas, a fim de proporcionar uma melhor compreensão na sequência lógica das cenas, incluindo: abordagem conceitual e fatores de risco acerca do extravasamento para enfermeiros; acolhimento, instalação do esquema quimioterápico e ações de prevenção; orientações e manifestações clínicas do extravasamento e manejo adequado frente à ocorrência de extravasamento. Além disso, o vídeo disponibiliza legendas como uma ferramenta tecnológica adicional, destinada a aprimorar a compreensão do público-alvo, contribuindo assim para otimizar a prestação de cuidados de enfermagem de alta qualidade.

O vídeo educativo, intitulado Prevenção e manejo no extravasamento de agentes antineoplásicos está disponível para acesso *online* em: (https://www.youtube.com/watch?v=02pGCHrndns). Além disso, o vídeo disponibiliza legendas como uma ferramenta tecnológica adicional, destinada a aprimorar a compreensão do público-alvo, contribuindo assim para otimizar a prestação de cuidados de enfermagem de alta qualidade.

Os juízes envolvidos na validação do conteúdo do roteiro e *storyboard* do vídeo educativo eram todos profissionais graduados em enfermagem, com título de doutorado e experiência prévia comprovada na administração de drogas antineoplásicas. Eles avaliaram o roteiro e *storyboard,* realizando sugestões nas questões referentes à relevância e ambiente. Destacaram que algumas terminologias e imagens deveriam ser trocadas ou complementadas, pois eram consideradas inadequadas.

O IVC médio e geral, resultante da avaliação, demonstrou-se superior ao limite mínimo de concordância estipulado para este estudo. Especificamente, o IVC geral alcançou 90,8%, reafirmando a significativa contribuição do vídeo educativo para o enriquecimento da área de conhecimento (Tabela 1).

**Tabela 1:** Distribuição de frequência absoluta do nível de concordância dos juízes de avaliação de conteúdo (n = 9) e Índice de Validade de Conteúdo de cada domínio do instrumento de validação do roteiro e *storyboard* do vídeo educativo, Ribeirão Preto, São Paulo, Brasil, 2024

Domínio	D^†^	DP^‡^	I^§^	CP^ǁ^	C^¶^	IVC^*^(%)
Objetivos						
Os objetivos estão coerentes com a prática de enfermagem.	-	-	-	1	8	100
Os objetivos estão adequados para serem atingidos.	-	-	-	-	9	100
IVC Médio	-	-	-	-	-	100
Conteúdo						
O conteúdo apresentado no roteiro e *storyboard* corresponde aos objetivos propostos.	-	-	-	3	6	100
O conteúdo facilita o processo ensino-aprendizagem acerca da prevenção e manejo no extravasamento de agentes antineoplásicos.	-	-	-	2	7	100
O conteúdo permite a compreensão da prevenção e manejo no extravasamento de agentes antineoplásicos.	-	-	-	3	6	100
O conteúdo obedece a uma sequência lógica.	-	-	-	2	7	100
O conteúdo incorpora os passos necessários para a realização da prevenção e manejo no extravasamento de agentes antineoplásicos.	-	-	-	4	5	100
O conteúdo dispõe dos materiais necessários para demonstração da prevenção e manejo no extravasamento de agentes antineoplásicos.	-	-	-	1	8	100
As informações do roteiro e *storyboard* estão corretas.	1	-	-	3	5	89
IVC Médio	-	-	-	-	-	98,4
Relevância						
As imagens e cenas ilustram aspectos importantes para a prática de prevenção e manejo no extravasamento de agentes antineoplásicos.	-	-	2	2	5	78
As imagens e cenas são relevantes para que o enfermeiro possa compreender a prevenção e manejo no extravasamento de agentes antineoplásicos.	-	-	1	2	6	89
As imagens e cenas permitem transferência e utilização do conhecimento teórico/prático em diferentes contextos pelos enfermeiros.	-	-	2	1	6	78
IVC Médio	-	-	-	-	-	81,6
Ambiente						
O cenário é adequado para a produção do vídeo.	-	-	2	2	5	78
O cenário é adequado para o ensino e aprendizagem acerca da prevenção e manejo no extravasamento de agentes antineoplásicos.	-	-	1	2	6	89
IVC Médio	-	-	-	-	-	83,5
IVC GERAL	-	-	-	-	-	90,8

* *- Índice de Validade de Conteúdo;* **† -** *Discordo;* **‡ -** *Discordo parcialmente;* **§ -** *Indiferente;* ǁ - *Concordo parcialmente;* **¶ -** *Concordo.*

Entre os juízes de avaliação técnica, todos atuavam na área de comunicação e tecnologias audiovisuais com ênfase na área da saúde e enfermagem, sendo um com título de mestrado, três com especialização e um com graduação. Na validação de técnica do roteiro e *storyboard*, houve concordância de 100% no domínio relacionado a funcionalidade. Os domínios usabilidade e eficiência, obteve concordância mínima de 88%, sendo justificado pelos juízes que a linguagem visual poderia ajudar a esclarecer melhor alguns pontos e tornar o conteúdo mais dinâmico. No aspecto relacionado à eficiência, um juiz ressaltou a necessidade de incluir mais esquemas quimioterápicos no vídeo, uma sugestão que, porém, não foi acolhida devido à limitação de duração recomendado para vídeos educativos.

O IVC médio e geral, atingiu o nível de concordância mínimo exigido, apresentando um IVC geral de 94,2%, conforme observado na Tabela 2.

**Tabela 2:** Distribuição de frequência absoluta do nível de concordância dos juízes de avaliação técnica em vídeo (n = 5) e Índice de Validade de Conteúdo de cada domínio do instrumento de validação do roteiro e *storyboard* do vídeo educativo, Ribeirão Preto, São Paulo, Brasil, 2024

Domínio	D^†^	DP^‡^	I^§^	CP^ǁ^	C^¶^	IVC^*^(%)
Funcionalidade						
O roteiro e *storyboard* do vídeo propõe intervenções de enfermagem compreensíveis para a prevenção e manejo no extravasamento de agentes antineoplásicos.	-	-	-	3	2	100
O roteiro e *storyboard* do vídeo tem potencial para gerar resultados positivos.	-	-	-	-	5	100
IVC Médio	-	-	-	-	-	100
Usabilidade						
É fácil de aprender os conceitos que serão utilizados no vídeo e suas aplicações.	-	1	-	2	2	80
O vídeo permitirá que o enfermeiro aprenda as intervenções prevenção e manejo no extravasamento de agentes antineoplásicos.	-	-	-	2	3	100
O vídeo auxiliará o enfermeiro de forma clara e eficiente, não sendo cansativo.	-	1	-	1	3	80
IVC Médio	-	-	-	-	-	86,7
Eficiência						
O tempo proposto é adequado para que o usuário aprenda o conteúdo.	-	-	-	2	3	100
O número de cenas está coerente com o tempo proposto para o vídeo.	-	-	-	2	3	100
O número e a caracterização dos personagens atendem ao objetivo proposto.	-	-	-	1	4	100
A comunicação entre os personagens ocorre de forma eficiente e compreensível.	-	-	-	3	2	100
A descrição dos materiais que serão utilizados está clara.	1	-	-	1	3	80
IVC Médio	-	-	-	-	-	96
IVC GERAL	-	-	-	-	-	94,2

* *- Índice de Validade de Conteúdo; † - Discordo; ‡ - Discordo parcialmente; § - Indiferente;* ǁ *- Concordo parcialmente; ¶ - Concordo.*

No que concerne a avaliação do vídeo educativo pelo público-alvo, um total de 20 enfermeiros foram convidados a participar, dos quais onze aceitaram o convite, sendo sete profissionais que atuavam em ambulatório de quimioterapia, três em unidade de internação oncológica e um em Unidade de Transplante de Medula Óssea (TMO), com tempo de atuação profissional de um a 20 anos e média de nove anos. Quanto à titulação acadêmica nove possuíam especialização, um doutorado e um apenas graduação.

A Tabela 3 demonstra os aspectos da avaliação do vídeo educativo pelo público-alvo, apontando que a maioria avaliou o vídeo de modo positivo. Apenas um profissional indicou o item “não”.

**Tabela 3:** Distribuição da resposta do público-alvo referente a avaliação do vídeo educativo, Ribeirão Preto, São Paulo, Brasil, 2023

Variável	Sim		Não	
	f	%	f	%
O conteúdo do vídeo apresenta informações importantes para a prática da prevenção e manejo no extravasamento de agentes antineoplásicos?	11	100	-	-
O conteúdo do vídeo está claro e compreensível?	11	100	-	-
As imagens e cenas auxiliam para a compreensão do extravasamento de agentes antineoplásicos?	11	100	-	-
Assistir ao vídeo auxilia na compreensão do extravasamento de agentes antineoplásicos?	11	100	-	-
O tempo do vídeo é adequado?	10	90,9	1	9,1

Os participantes avaliaram o vídeo de maneira positiva, referindo que o conteúdo do vídeo apresenta informações significativas, clareza e uma linguagem que favorece a compreensão da temática. As sugestões fornecidas pelos participantes foram avaliadas e incorporadas conforme apropriado. Consequentemente, o vídeo foi validado com o potencial de contribuir de maneira significativa para o ensino da temática proposta, como também indicado pelas opiniões dos juízes de avaliação de conteúdo e de técnica.

## DISCUSSÃO

Todos os profissionais da equipe de oncologia partilham a responsabilidade de assegurar a administração segura da terapia quimioterápica^(9)^. No entanto, a equipe de enfermagem encontra-se sujeita a uma demanda assistencial mais intensa devido às potenciais injúrias resultantes do extravasamento desses agentes. Consequentemente, é imperativo que os profissionais de enfermagem não apenas estejam cientes dos riscos inerentes, mas também possuam habilidade técnica, compreendam a importância desses procedimentos e evitem distrações e interrupções durante a administração da terapia quimioterápica^(6-8)^.

A elaboração de recursos educativos adicionais, como o vídeo, emerge como uma abordagem que fortalece a integração de intervenções pautadas em evidências científicas, ao mesmo tempo que oferece uma solução tecnológica que aprimora a educação em saúde. O vídeo, dotado de características atrativas e acessibilidade ampliada, devido à fácil disponibilidade, é capaz de alcançar vasta abrangência geográfica, reforçando sua utilidade como meio eficaz de disseminação de conhecimento^(20)^.

Evidencia-se, portanto, a relevância intrínseca desse estudo, com a criação de um vídeo educativo, a fim de fornecer um recurso fundamental para enriquecer a capacitação de enfermeiros na assistência à pessoa com câncer, com foco na prevenção e manejo do extravasamento de agentes antineoplásicos. Assim, no desenvolvimento do vídeo educativo, uma preocupação notável foi dirigida à criação de um ambiente simulado, assemelhando-se aos cenários e recursos materiais encontrados em serviços de quimioterapia ambulatorial, incluindo um espaço privativo para a assistência à pessoa com câncer. Estudos na área da enfermagem têm revelado a crescente utilização da simulação clínica como metodologia ativa de ensino no âmbito da saúde^(21-23)^.

A produção de um vídeo educativo é um processo rigoroso, de longa duração e intenso, demandando empenho dos pesquisadores. Seu objetivo é criar uma ferramenta enriquecida com intervenções embasadas cientificamente, que promovam a construção de conhecimento de acordo com as necessidades dos profissionais de enfermagem. Esse empreendimento segue uma abordagem rigorosa, garantindo a construção de um material educativo digital sólido e embasado em evidências^(24)^.

No presente estudo, houve o desenvolvimento do vídeo educativo a partir da elaboração de um roteiro e *storyboard*, tendo como base um caso clínico fictício que integra a experiência profissional do pesquisador principal. Além disso, foram investigadas as lacunas de conhecimento^(6-8)^ da assistência de enfermagem na prevenção e manejo do extravasamento de agentes antineoplásicos.

A utilização de roteiro e *storyboard* no desenvolvimento de vídeo educativo é uma abordagem que abrange a organização sequencial do conteúdo a ser produzido, incluindo a análise do cenário, recursos e materiais disponíveis. Essa estratégia visa especificar detalhes como narração, imagens e cenas envolvidas na produção de vídeos educativos^(25)^.

Estudos têm revelado a utilização de materiais digitais educativos, como o vídeo no contexto da enfermagem, com a construção antecipada do roteiro e *storyboard*, semelhante à produção realizada neste estudo. Esses estudos demonstram que a utilização de roteiro e *storyboard* torna o processo de criação mais estruturado, facilitando a compreensão do público-alvo sobre a temática. Tais pesquisas têm se apoiado em casos clínicos vivenciados nos diversos cenários de saúde, fundamentados no conhecimento para a criação dos materiais educativos^(20,26-28)^.

A validação de um roteiro e *storyboard* requer a seleção criteriosa de juízes na temática em estudo. Neste estudo, foram adotados os critérios estabelecidos por Fehring^(29)^, uma abordagem amplamente reconhecida que visa a utilidade do instrumento para aplicação na prática em saúde.

A escolha dos juízes de conteúdo através da Plataforma Lattes do CNPq é uma estratégia frequentemente utilizada pelos pesquisadores, permitindo identificar profissionais atuantes em diferentes realidades de saúde do país. Essa abordagem contribui para agregar perspectivas valiosas, especialmente em relação às nuances da assistência em diferentes contextos locais de interesse^(26,30,31)^.

A validação do roteiro e *storyboard* alcançou o nível de concordância mínimo exigido para este estudo, revelando um resultado semelhante aos estudos de desenvolvimento de vídeos educativos^(20,26-28)^. A avaliação do vídeo pelo público-alvo foi fundamental para confirmar a validação feita pelos juízes especialistas.

Embora tenham sido apresentadas sugestões de alteração no roteiro, *storyboard* e no próprio vídeo por parte dos juízes de avaliação de conteúdo e de técnica e público-alvo, essas considerações resultaram em melhorias na coerência dos elementos do vídeo. Como resultado, o vídeo educativo foi validado como uma valiosa ferramenta para enriquecer o ensino da temática proposta.

Contudo, mesmo após todo o processo de validação, gravação, edição e finalização do vídeo, algumas fragilidades foram identificadas, tais como: o uso de avental comum (do tipo isolamento de contato), quando o recomendado é o avental impermeável com punhos firmes por não permitir a exposição da pele^(16)^; a não permanência do profissional em posição postural durante a infusão de quimioterápicos vesicantes, não sendo capaz de avaliar o local de punção e as manifestações clínicas do extravasamento e emocionais do paciente^(16)^, e, por fim, a utilização de um único par de luvas, sendo que as evidências aconselham o uso de dois pares de luvas de procedimento, uma vez que há micro orifícios neste tipo de luva e uma sobre a outra evitaria a principal via de contaminação para o profissional, que é a dermatológica^(32)^.

A versão final do vídeo educativo excedeu a recomendação de duração de 15 minutos, o que pode suscitar distração do telespectador, de acordo com estudos anteriores^(25,33)^. No entanto, há divergências sobre o limite ideal de duração para vídeos educativos voltados para a saúde, variando entre 15 a 20 minutos^(34)^, o que gera questionamentos sobre a recomendação a seguir.

Em suma, o vídeo educativo foi disponibilizado na rede, por meio de uma plataforma digital, possibilitando a comunicação audiovisual na Internet, conforme proposto por Falkembach^(15)^, através da fase de distribuição, sendo a escolha deste ambiente justificada pela facilidade de acesso e interesse em reforçar, de forma didática, orientações fornecidas aos profissionais de enfermagem acerca da temática.

### Limitações do estudo

Apesar dos resultados positivos e da avaliação do vídeo educativo, é importante mencionar algumas limitações deste estudo, como o tamanho do roteiro e *storyboard*, que pode ter impactado a avaliação dos especialistas, a duração final do vídeo, a falta de legendas em outros idiomas e recursos para ensinar Libras a pessoas com deficiência auditiva. No entanto, a avaliação positiva do vídeo é encorajadora. Para futuros estudos, recomenda-se a aplicação em pesquisas experimentais para avaliar sua eficácia e uso na prática clínica de enfermagem como ferramenta de educação em saúde. Esse é um novo objetivo para investigações subsequentes.

### Contribuições para a área da enfermagem

Este estudo oferece uma valiosa contribuição ao proporcionar uma ferramenta educativa inovadora para profissionais de enfermagem, abordando a temática de forma acessível em plataformas digitais de livre acesso. Isso tem o potencial de beneficiar a assistência à pessoa com câncer, superando barreiras geográficas. A pesquisa destaca como as tecnologias podem impulsionar o progresso do conhecimento no campo da enfermagem, desempenhando papéis cruciais tanto na educação quanto na assistência.

## CONCLUSÕES

A construção, validação e avaliação do vídeo educativo representaram uma abordagem eficaz e bem recebida tanto pelos juízes de conteúdo e técnicos quanto pelo público-alvo. O vídeo demonstrou ser uma estratégia adequada para instruir intervenções acerca da prevenção e manejo do extravasamento de agentes antineoplásicos, com potencial para melhorar as práticas educativas entre profissionais de enfermagem. Os resultados alcançaram os objetivos estabelecidos e apresentaram índices adequados de validação. Ademais, esse processo destacou o empoderamento das práxis em enfermagem na criação de materiais educativos digitais efetivos, revelando novas perspectivas na organização e gestão do cuidado.
